# Supplementary figures and images for: The Vibrio cholerae Seventh Pandemic Islands act in tandem to defend against a circulating phage
Source: PLoS Genet. 2022 Aug 26;18(8):e1010250. doi: 10.1371/journal.pgen.1010250 (PMC9455884; doi:10.1371/journal.pgen.1010250)

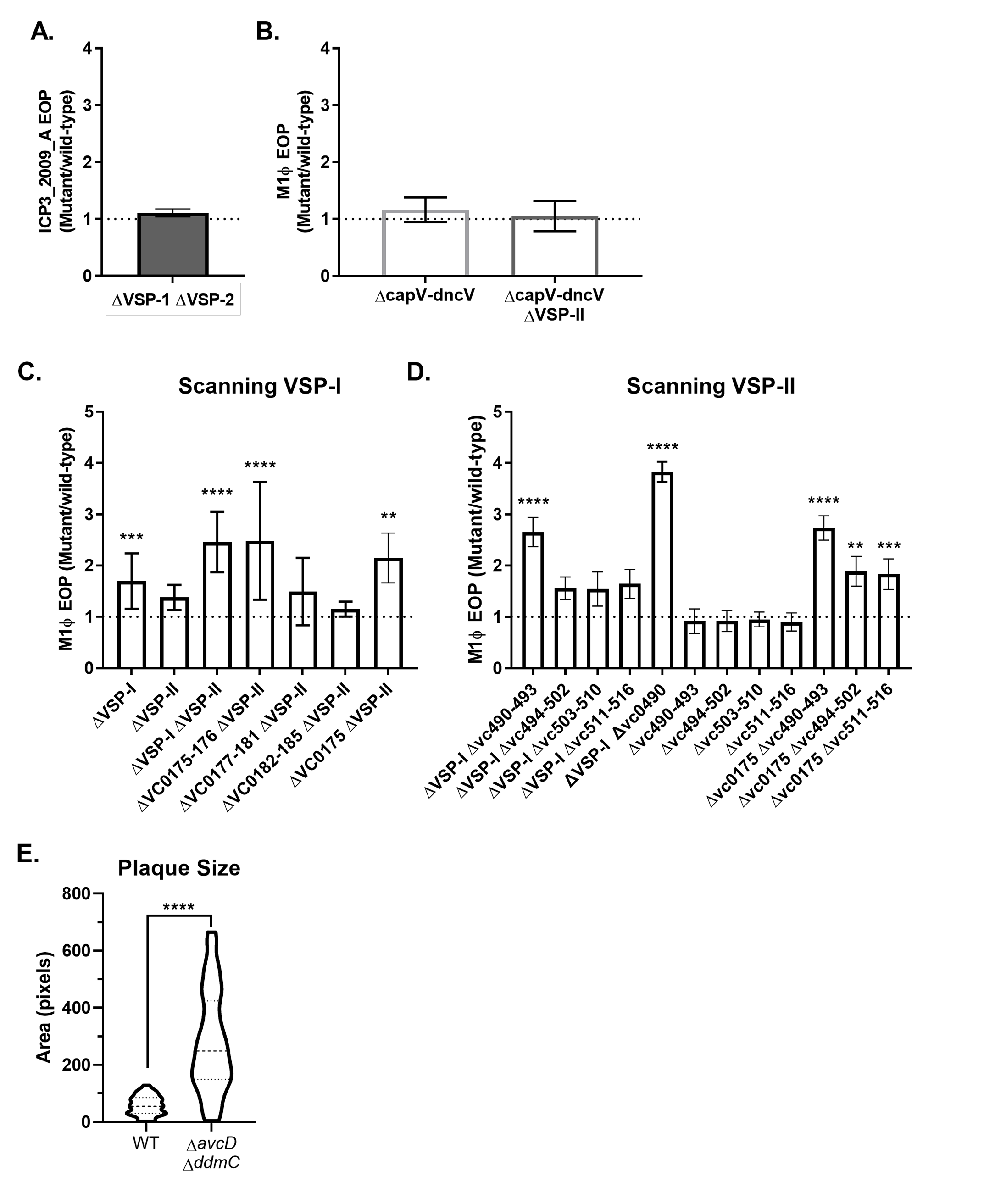

Supplement: S1 Fig — (A) EOP of ICP3_2009_A infection determined on the V. cholerae ΔVSP-I ΔVSP-II mutant and the parental VSP+ strain (WT). (B) EOP of ICP3_2016_M1 (M1Φ) infection determined on the capV-dncV mutant and WT. (C) EOP of M1Φ infection determined on different VSP-I related mutants and WT. Values from ΔVSP-I, ΔVSP-II, and ΔVSP-I VSP-II are also shown in Fig 2 and repeated here for clarity. (D) EOP of M1Φ infection determined on different VSP-II related mutants and WT. (E) Quantification of plaque sizes. Plaques were imaged and processed with Fiji to determine the area of each plaque on a plate. Sizes are from plates from two separate days. Significance was determined by Kolmogorov–Smirnov test where **** = p<0.0001. For EOP assays significance was determined by one-way ANOVA with Dunnett’s multiple comparison test comparing the EOP of each strain to the control of the WT strain. Signifiers for significance were displayed based on p-values as follows: 0.1234(ns), 0.0332(*), 0.0021(**), 0.0002(***), <0.0001(****). (TIF) [file pgen.1010250.s001.tif]

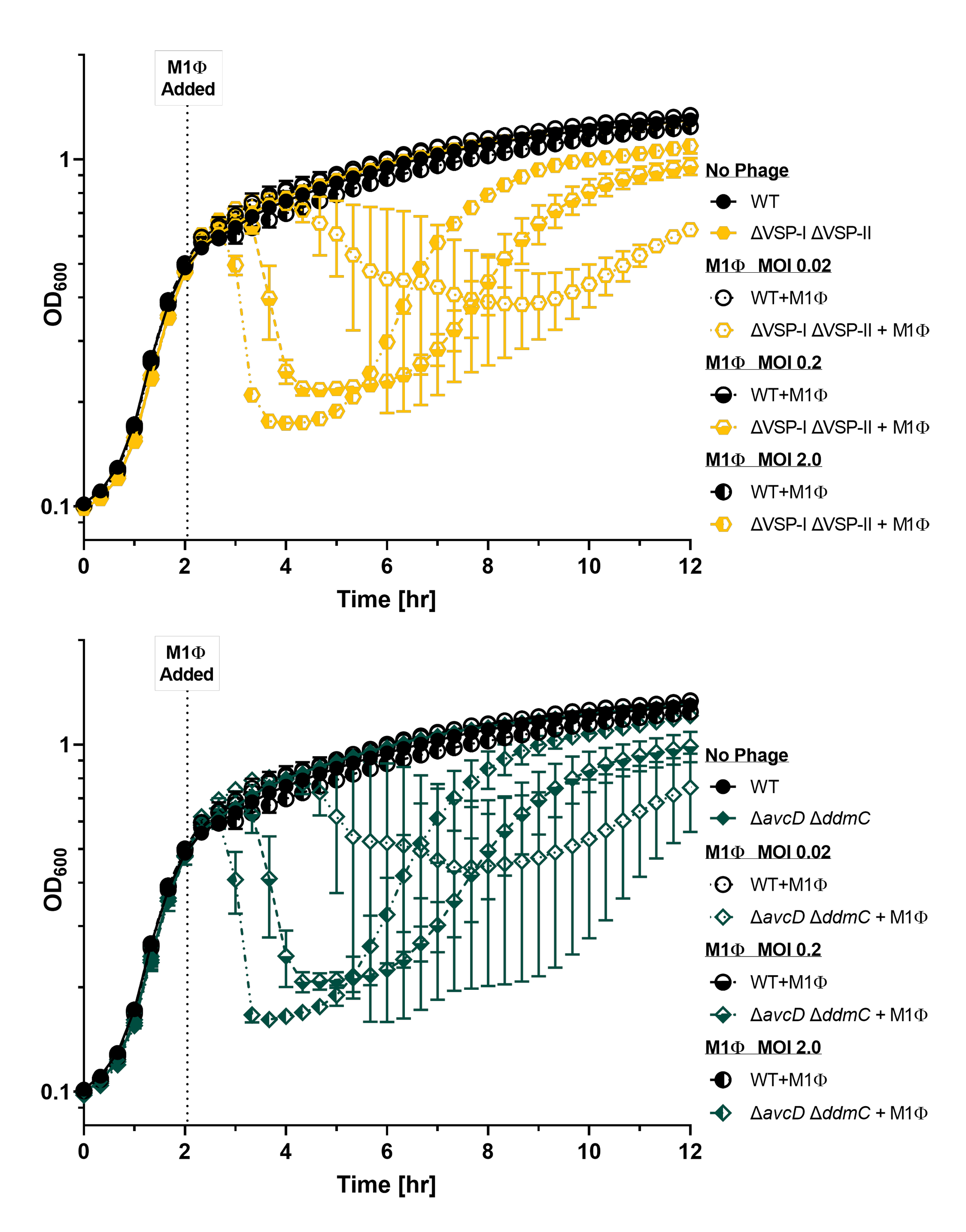

Supplement: S2 Fig — Growth and lysis curves of V. cholerae when infected at with varied MOI of phage. Indicated strains were infected with phage at the time shown with a dotted line (OD~0.5). Both graphs come from the same dataset and are shown separately for clarity. WT data is therefore identical for these two graphs. (TIF) [file pgen.1010250.s002.tif]

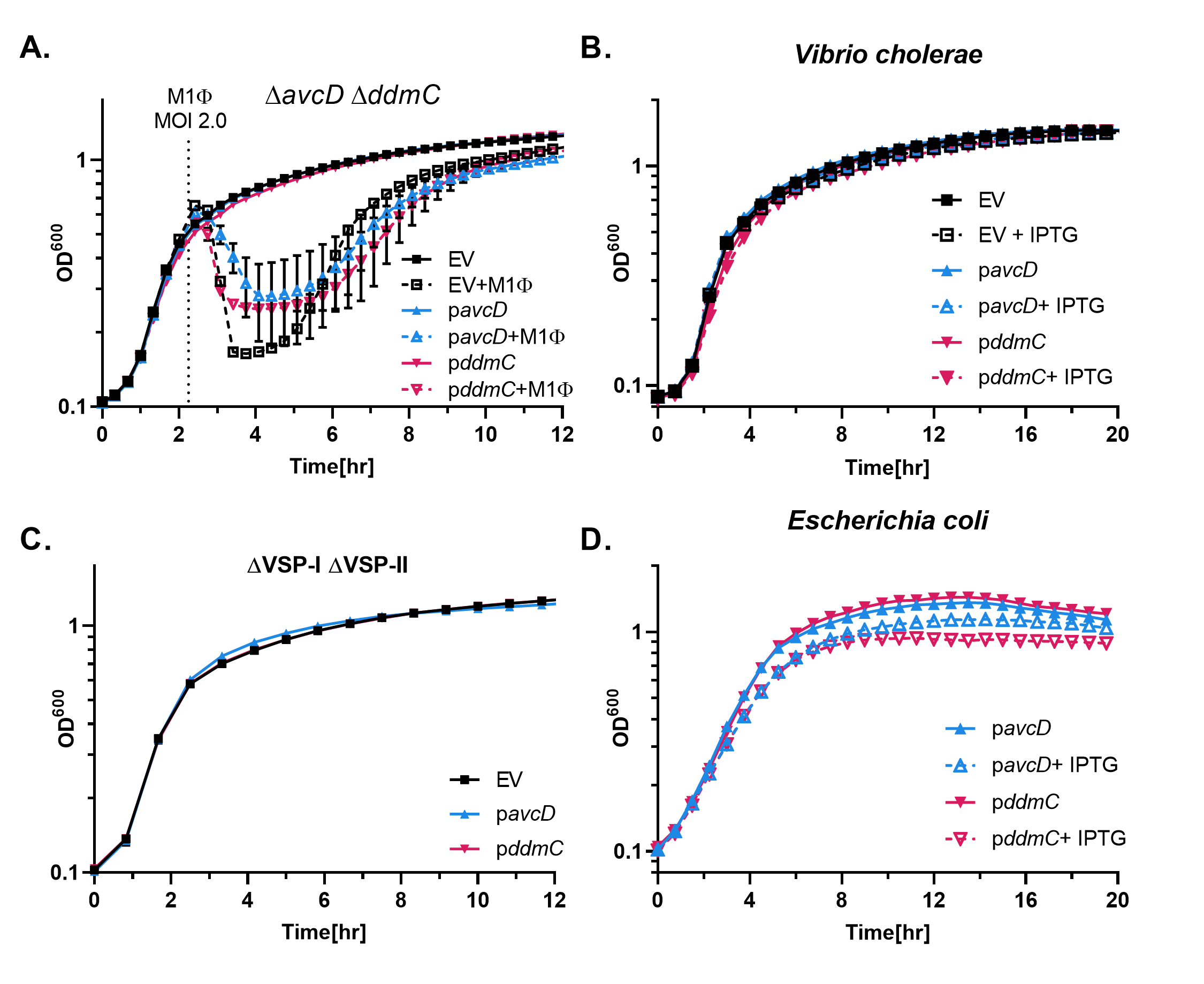

Supplement: S3 Fig — Growth curves of V. cholerae (A) and E. coli (B) carrying the plasmid pMMB67eh with the indicated insert +/- 100μM IPTG. Average of at least three biological replicates are shown. (C) V. cholerae ΔavcD ΔddmC strain containing the plasmid pMMB67eh or the same plasmid expressing avcD or ddmC was grown and infected with M1Φ at an MOI of 2. All strains induced with 100μM IPTG (D) ΔVSP-I ΔVSP-II V. cholerae containing the plasmid pMMB67eh with the indicated insert were grown in inducing conditions with 100μM IPTG. (TIF) [file pgen.1010250.s003.tif]

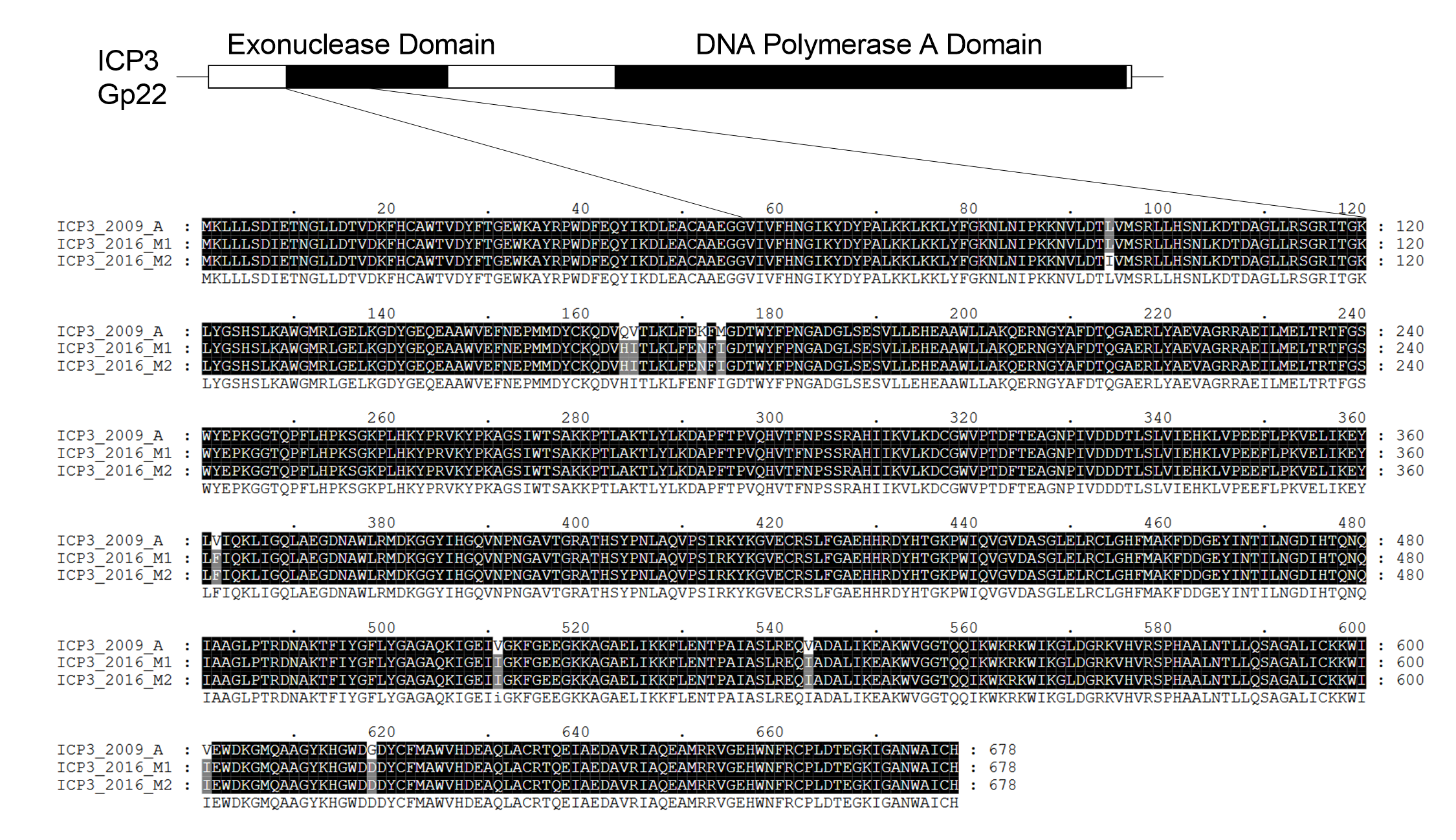

Supplement: S4 Fig — Scale diagram of the ICP3 polymerase GP22 with the black boxes indicating the well conserved exonuclease and DNA Polymerase A domains. Breakout alignment of M1 and M2 gp22 was performed with T-Coffee and display was generated using GENEDOC. Single amino acid change can be seen as black text on a grey background. (TIF) [file pgen.1010250.s004.tif]
