# Supplementary material for: The Vibrio cholerae Seventh Pandemic Islands act in tandem to defend against a circulating phage
Source: PLoS Genet. 2022 Aug 26;18(8):e1010250. doi: 10.1371/journal.pgen.1010250 (PMC9455884; doi:10.1371/journal.pgen.1010250)
Supplement: S1 Table — (DOCX) [file pgen.1010250.s005.docx]

**Supplemental Table 1: Summary of phage found in stool samples**

|  | **ICP1** | **ICP2** | **ICP3** | **No Bands** |
| --- | --- | --- | --- | --- |
| Sample 1 | **ND** | **ND** | **ND** | **N/A** |
| Sample 2 | **93.75%** | **ND** | **ND** | **6.25%** |
| Sample 3 | **12.50%** | **12.50%** | **37.50%** | **37.50%** |
| Sample 4 | **ND** | **ND** | **ND** | **N/A** |

Picked plaques were boiled, diluted, and used as template for diagnostic PCRs using primers targeting the conserved polymerases of ICP1, ICP2, and ICP3. Percent of plaques with indicated band shown. No Bands indicates no bands were detected after two separate attempts to amplify the sample. ND = none detected. N/A = not applicable as no plaques were recovered from these samples after successive attempts.
