# Supplementary material for: The Vibrio cholerae Seventh Pandemic Islands act in tandem to defend against a circulating phage
Source: PLoS Genet. 2022 Aug 26;18(8):e1010250. doi: 10.1371/journal.pgen.1010250 (PMC9455884; doi:10.1371/journal.pgen.1010250)
Supplement: S2 Table — (DOCX) [file pgen.1010250.s006.docx]

**Supplemental Table 2: Bacterial strains used in this study**

| **Strain #** | **Background** | | **Description** | | **Source** |  |
| --- | --- | --- | --- | --- | --- | --- |
| WN6145 | *V. cholerae* E7946 | SmR *V. cholerae* O1, El Tor Biotype;  ΔRSI-CTX-TLC, ΔK139-*att*B, *wbeL*::A111G+A114G *manA*::A216G,A219G,A609G,A612G.  O1-locked strain with Kappa and CTX phage deleted. Used as the VSP^+^ parental strain for most phage experiments | | Camilli lab | | |
| WN001 | *V. cholerae* C6706 | SmR *V. cholerae* O1, El Tor Biotype; used as WT in quorum sensing experiments. | | [6] | | |
| WN7006 | *V. cholerae* WN6145 | SmR, SpecR, ΔVSP-II (∆*vc0490-vc0516*) ΔVSP-I::spec (∆*vc0175-0185*::spec) | | This study | | |
| WN7007 | *V. cholerae* WN6145 | SmR, SpecR, ΔVSP-I::spec | | This study | | |
| WN7008 | *V. cholerae* WN6145 | SmR, SpecR, Δ*vc0175* Δ*lacZ*::specR | | This study | | |
| WN7009 | *V. cholerae* WN6145 | SmR, SpecR, Δ*vc0175* Δ*lacZ*::specR ΔVSP-II | | This study | | |
| WN7010 | *V. cholerae* WN6145 | SmR, ΔVSP-II | | This study | | |
| WN7011 | *V. cholerae* WN6145 | SmR, SpecR, Δ*vc0490* Δ*lacZ*::specR | | This study | | |
| WN7012 | *V. cholerae* WN6145 | SmR, KanR, Δ*vc0175* Δ*vc0490* Δ*lacZ*::kanR | | This study | | |
| WN7013 | *V. cholerae* WN6145 | SmR, AmpR, KanR, pMMB67EH::*vc0175* | | This study | | |
| WN7014 | *V. cholerae* WN6145 | SmR, AmpR, KanR, pMMB67EH::EV | | This study | | |
| WN7015 | *V. cholerae* WN6145 | SmR, AmpR, KanR, pMMB67EH::*vc0175* Δ*vc0175* Δ*vc0490* Δ*lacZ*::kanR | | This study | | |
| WN7016 | *V. cholerae* WN6145 | SmR, AmpR, KanR, pMMB67EH::EV Δ*vc0175* Δ*vc0490* Δ*lacZ*::kanR | | This study | | |
| WN7017 | *V. cholerae* WN6145 | SmR, AmpR, pMMB67eh::*vc0490* | | This study | | |
| WN7018 | *V. cholerae* WN6145 | SmR, AmpR, SpecR, pMMB67eh::*vc0490* Δ*vc0175* Δ*vc0490* Δ*lacZ*::specR | | This study | | |
| WN7019 | *V. cholerae* WN6145 | SmR, SpecR, Δ*lacZ*::specR | | This study | | |
| WN7020 | *V. cholerae* WN6145 | SmR, SpecR, Δ*vc0175* Δ*vc0490* Δ*lacZ*::specR | | This study | | |
| WN7021 | *V. cholerae* WN001 | SmR, CmR, pBBRlux::*vc0492* | | This study | | |
| WN7022 | *V. cholerae* WN001 | SmR, CmR, pBBRlux::*vc0492* Δ*luxO* | | This study | | |
| WN7023 | *V. cholerae* WN001 | SmR, CmR, pBBRlux::*vc0492* Δ*hapR* | | This study | | |
| WN7024 | *V. cholerae* WN001 | SmR, CmR, pBBRlux::*vc0492* Δ*luxS* Δ*cqsA* | | This study | | |
| WN072 | *V. cholerae* WN001 | SmR, Δ*luxO* | | [4] | | |
| WN744 | *V. cholerae* WN001 | SmR, Δ*cqsA* Δ*luxS* | | [3] | | |
| WN7025 | *V. cholerae* WN6145 | SmR, Δ*capV* Δ*dncV* | | This study | | |
| WN7026 | *V. cholerae* WN6145 | SmR, ΔVSP-II Δ*capV-dncV* | | This study | | |
| WN7027 | *V. cholerae* WN6145 | SmR, SpecR, Δ*vc0175-176*::spec ΔVSP-II | | This study | | |
| WN7028 | *V. cholerae* WN6145 | SmR, SpecR, Δ*vc0177-181*::spec ΔVSP-II | | This study | | |
| WN7029 | *V. cholerae* WN6145 | SmR, SpecR, Δ*vc0182-185*::spec ΔVSP-II | | This study | | |
| WN7030 | *V. cholerae* WN6145 | SmR, KanR, SpecR, Δ*vc0490-493*::kan ΔVSP-I::spec | | This study | | |
| WN7031 | *V. cholerae* WN6145 | SmR, KanR, SpecR, Δ*vc0494-502*::kan ΔVSP-I::spec | | This study | | |
| WN7032 | *V. cholerae* WN6145 | SmR, KanR, SpecR, Δ*vc0503-510*::kan ΔVSP-I::spec | | This study | | |
| WN7033 | *V. cholerae* WN6145 | SmR, KanR, SpecR, Δ*vc0511-51*6::kan ΔVSP-I::spec | | This study | | |
| WN7034 | *V. cholerae* WN6145 | SmR, SpecR, KanR, ΔVSP-I::spec Δ*vc0490* Δ*lacZ*::kanR | | This study | | |
| WN7035 | *V. cholerae* WN6145 | SmR, KanR, Δ*vc0490-493*::kan | | This study | | |
| WN7036 | *V. cholerae* WN6145 | SmR, KanR, Δ*vc0494-502*::kan | | This study | | |
| WN7037 | *V. cholerae* WN6145 | SmR, KanR, Δ*vc0503-510*::kan | | This study | | |
| WN7038 | *V. cholerae* WN6145 | SmR, KanR, Δ*vc0511-516*::kan | | This study | | |
| WN7039 | *V. cholerae* WN6145 | SmR, KanR, SpecR, Δ*vc0175* Δ*vc0490-493*::kan Δ*lacZ*::specR | | This study | | |
| WN7040 | *V. cholerae* WN6145 | SmR, KanR, SpecR, Δ*vc0175* Δ*vc0494-502*::kan Δ*lacZ*::specR | | This study | | |
| WN7041 | *V. cholerae* WN6145 | SmR, KanR, SpecR, Δ*vc0175* Δ*vc0511-516*::kan Δ*lacZ*::specR | | This study | | |
| WN6493 | *E. coli* S17 | pMMB67EH::*vc0175* | | [5] | | |
| WN7042 | *E. coli* S17 | pMMB67eh::*vc0490* | | This study | | |
| WN5682 | *E. coli* S17 | pMMB67EH::EV | | [2] | | |
| WN0479 | *E. coli* S17 | pEVS143::EV | | [1] | | |
| WN6029 | *E. coli* S17 | AmpR pKas32::Δ*capV-dncV* | | This Study | | |
| WN6147 | *E. coli* BW29427 | AmpR pKas32::ΔVSP-II (=pCCR02) | | Waters Lab | | |

REFERENCES:

1. Dunn AK, Millikan DS, Adin DM, Bose JL, Stabb E V. New rfp- and pES213-derived tools for analyzing symbiotic Vibrio fischeri reveal patterns of infection and lux expression in situ. Appl Environ Microbiol. 2006;72: 802–810. doi:10.1128/AEM.72.1.802-810.2006
2. Fürste JP, Pansegrau W, Frank R, Blöcker H, Scholz P, Bagdasarian M, et al. Molecular cloning of the plasmid RP4 primase region in a multi-host-range tacP expression vector. Gene. 1986;48: 119–131. doi:10.1016/0378-1119(86)90358-6
3. Hammer BK, Bassler BL. Regulatory small RNAs circumvent the conventional quorum sensing pathway in pandemic Vibrio cholerae. Proc Natl Acad Sci U S A. 2007;104: 11145–11149. doi:10.1073/pnas.0703860104
4. Hammer BK, Bassler BL. Distinct sensory pathways in Vibrio cholerae El Tor and classical biotypes modulate cyclic dimeric GMP levels to control biofilm formation. J Bacteriol. 2009;91: 169–177. doi:10.1128/JB.01307-08
5. Hsueh BY, Severin GB, Elg CA, Waldron EJ, Kant A, Wessel AJ, et al. Phage defence by deaminase-mediated depletion of deoxynucleotides in bacteria. Nat Microbiol. 2022. doi:10.1038/s41564-022-01162-4
6. Thelin K, Taylor RK. Toxin-coregulated pilus, but not mannose-sensitive hemagglutinin, is required for colonization by Vibrio cholerae O1 El Tor biotype and O139 strains. Infect Immun. 1996;64: 2853–2856. doi:10.1128/iai.64.7.2853-2856.1996
