# Supplementary material for: The Vibrio cholerae Seventh Pandemic Islands act in tandem to defend against a circulating phage
Source: PLoS Genet. 2022 Aug 26;18(8):e1010250. doi: 10.1371/journal.pgen.1010250 (PMC9455884; doi:10.1371/journal.pgen.1010250)
Supplement: S3 Table — (DOCX) [file pgen.1010250.s007.docx]

| **S3 TABLE: Oligonucleotides used in this study** | | |
| --- | --- | --- |
| **Name** | **Description** | **Sequence** |
| WNTP1309 | *vc0175* F0 | ccctgatttcagtcacagag |
| WNTP1138 | *vc0175* R0 | tttctgactttggctccgcatg |
| WNTP1589 | *vc0175* F1 | tccagactaaagctacaacctgaaattatgaaact |
| WNTP1588 | *vc0175* R1 | ataatttcaggttgtagctttagtctggaaaattcac |
| WNTP1308 | VSP-I SpecR R1 | gcaaacgacgaaaactagaatcatgccgaaaaactttac |
| WNTP0516 | VSP-I SpecR F2 | tagttttcgtcgtttgctgcttataatttttttaatctgttatttaaatagtttatagttaaatttac |
| WNTP1139 | VSP-I SpecR R2 | gcgcaacgcaattaatgt |
| WNTP1305 | VSP-I SpecR F3 | cattaattgcgttgcgccgatctacatggtaacgaac |
| WNTP1306 | VSP-I SpecR R3 | ggagaaagtaatggcaacac |
| WNTP1621 | *vc0490* F0 | gaagcaccttggcgtgatgg |
| WNTP1553 | *vc0490* overlap R | aggttgttctttggtcataactggattggctataaagctaaacg |
| WNTP1552 | *vc0490* overlap R | ctttatagccaatccagttatgaccaaagaacaaccttttc |
| WNTP1693 | *vc0490* R0 | gataacaatttcacaatgaaagacaacagtacaagtg |
| WNTP0070 | *lacZ* F0 | gcgaccccaccgatggg |
| WNTP0071 | *lacZ* R0 | cccaaatacggcaacttggcg |
| WNTP1690 | pMMB67eh::*vc0490* F1 | gataacaatttcacaatgttttttcaaatagaaaagg |
| WNTP1689 | pMMB67eh::*vc0490* R1 | gaattctgtttcctgttatagccaatccagcggaa |
| WNTP1377 | Δvc0175-176::spec F2 | taattgcgttgcgctggctcataatcttgaagctc |
| WNTP0989 | Δvc0175-176::spec R2 | caccaccgccattcaaac |
| WNTP1379 | Δvc0177-181::spec F1 | ggagaaggaactgtacttaa |
| WNTP1380 | Δvc0177-181::spec R1 | gcagcaaacgacgaaaactaatgtatttctaatac |
| WNTP1381 | Δvc0177-181::spec F2 | taattgcgttgcgccttaggtatactaattcattc |
| WNTP1382 | Δvc0177-181::spec R2 | caaacacctagatctaccca |
| WNTP0896 | Δvc0182-185::spec F1 | cgtccatatcgctcagag |
| WNTP1378 | Δvc0182-185::spec R1 | aaacgacgaaaactagaatgaattagtatacctaa |
| WNTP1663 | Δvc490-493::kan F1 | gattgccccatgtaacgcgc |
| WNTP1664 | Δvc490-493::kan F2 | tgagacacaacgtgggacttttactctaatttacg |
| WNTP1665 | Δvc490-493::kan R2 | tgtcttgatgatctttaggc |
| WNTP1666 | Δvc494-502::kan F1 | aaatgttcttctagagcacc |
| WNTP1667 | Δvc494-502::kan R1 | taacactggcagagcacctatgttttgttaatgac |
| WNTP1668 | Δvc494-502::kan F2 | tgagacacaacgtgggcaattcctagtttctactc |
| WNTP1669 | Δvc494-502::kan R2 | gtatcgaccatcatggttcc |
| WNTP1670 | Δvc503-510::kan F1 | gtttaatgcggattgctgcg |
| WNTP1671 | Δvc503-510::kan R1 | taacactggcagagcacccatgagaactaagcatc |
| WNTP1672 | Δvc503-510::kan F2 | tgagacacaacgtgggctcctgtcggaatcaatgg |
| WNTP1673 | Δvc503-510::kan R2 | gcaacctgctctgtttctgc |
| WNTP1674 | Δvc511-516::kan F1 | tggctatctcgttgtaagcg |
| WNTP1675 | Δvc511-516::kan R1 | taacactggcagagctgctatagacatcgacaacc |
| WNTP1676 | Δvc511-516::kan F2 | tgagacacaacgtgggatgtaccaccaaaatagtcg |
| WNTP1677 | Δvc511-516::kan R2 | accatcactgacgaagatgc |
| WNTP1323 | KanR R | ccacgttgtgtctcaaaatc |
| WNTP1324 | KanR F | gctctgccagtgttacaacc |
| WNTP1325 | VSP-II Kan R1 | gtaacactggcagagcgtacctgacgaatttgggtc |
| WNTP1326 | VSP-II Kan F1 | catcaatataaactgggacc |
| WNTP1327 | VSP-II F0 (VC0489) | gagtatgctgcttattgagc |
| WNTP1328 | VSP-II Kan F2 | tgagacacaacgtggtagatagacgttggtagac |
| WNTP1329 | VSP-II Kan R2 | ctgaagcgtcagacttagtg |
| WNTP1330 | VSP-II R0 (vc517) | agcggttaatgaatttggtc |
| WNTP1338 | ICP1gp58F | aacgctgcttttccttttga |
| WNTP1339 | ICP1gp58R | cccagcattgaggacactt |
| WNTP1340 | ICP2gp50F | agcgttgtcattcgactgtg |
| WNTP1341 | ICP2gp50R | tcagcatgttctggacgaag |
| WNTP1342 | ICP3gp5F | attgtcgagtgggacaaagg |
| WNTP1343 | ICP3gp5R | accaactcgacgcatagctt |
| WNTP0911 | pMMB67eh linearization F | tgtgaaattgttatccgctc |
| WNTP0912 | pMMB67eh linearization R | caggaaacagaattcgag |
| WNTP0592 | Δ*capV-dncV F1* | gcgggtaccgcagatactaacaggtgatgg |
| WNTP0860 | Δ*capV-dncV* R1 | ctgctgattttttcttgcaccaccgccattcaaactaag |
| WNTP0861 | Δ*capV-dncV* F2 | ggtggtgcaagaaaaaatcagcagcacaatggtaagtgg |
| WNTP0862 | Δ*capV-dncV* R2 | aaggcggccgcttggcactcacaaacttgccacc |
| WN1338 | Vc0492-lux fusion (forward) | GGGACTAGTAAGAACTTCATCGTGAATAG |
| WN1339 | Vc0492-lux fusion (reverse) | AGTGGATCCtcaacctcttatgaaatcataccaa |
